# Impact of chronic obstructive pulmonary disease on the efficacy and safety of neoadjuvant immune checkpoint inhibitors combined with chemotherapy for resectable non-small cell lung cancer: a retrospective cohort study

**DOI:** 10.1186/s12885-024-11902-w

**Published:** 2024-01-30

**Authors:** Weigang Dong, Yan Yin, Shengnan Yang, Bin Liu, Xi Chen, Lina Wang, Yue Su, Yan Jiang, Dongsheng Shi, Daqiang Sun, Jianwen Qin

**Affiliations:** 1 Department of Respiratory and Critica Care Medicine, Tianjin Chest Hospital, Affiliated Chest Hospital of Tianjin University, Tianjin, China; 2 Department of Thoracic Surgery, Tianjin Chest Hospital, Affiliated Chest Hospital of Tianjin University, Tianjin, China

**Keywords:** COPD, Non-small cell lung cancer, Neoadjuvant, Immunochemotherapy

## Abstract

**Background:**

Neoadjuvant immune checkpoint inhibitors(ICIs) combined with chemotherapy can improve non-small cell lung cancer(NSCLC) patients' pathological responses and show promising improvements in survival. Chronic obstructive pulmonary disease (COPD) is a systemic inflammatory disease, and its associated abnormal inflammatory response affects not only the immunotherapy efficacy but also immune-related adverse events. It remains unclear whether NSCLC patients with COPD can benefit from neoadjuvant ICIs combined with chemotherapy.

**Methods:**

A retrospective observational clinical study was conducted on 105 consecutive NSCLC patients receiving neoadjuvant ICIs combined with chemotherapy at the Department of Thoracic Surgery of Tianjin Chest Hospital between April 2020 and April 2023.

**Results:**

A total of 74 NSCLC patients were included in the study, including 30 patients with COPD and 44 patients without COPD. The percentage of patients with a pathological complete response (PCR) was higher in the COPD group than in the non-COPD group (43.3% vs. 20.5%, *P* = 0.042). Multivariate logistic regression analysis of factors associated with PCR showed that the adjusted odds ratio (OR) was statistically significant for presence of COPD (OR = 3.020, 95%CI: 1.042–8.757; *P* = 0.042). Major pathological response (66.7% vs. 50%, *P* = 0.155), R0 resection rate (96.7% vs.93.2%, *P* = 0.642), N2 lymph node downstaging(92.3% vs. 66.7%, *P* = 0.182) and objective response rate (70% vs. 63.6%, *P* = 0.57) were not significantly different between the groups. Progression-free survival(PFS) was not reached in the COPD group and 17 months (95%CI: 12.1–21.9) in the non-COPD group, with statistically significance (χ^2^ = 6.247, *P* = 0.012). Multivariate Cox’s regression analysis showed that the adjusted hazard ratio (HRadj) was statistically significant for presence of COPD (HRadj = 0.321, 95%CI: 0.111–0.930; *P* = 0.036). The grade 3 and grade 4 adverse events in the COPD group were leukopenia (3.3%, 6.7%), neutropenia (3.3%, 6.7%), fatigue (6.7%, 0%), gastrointestinal reactions (3.3%, 0%), and hypothyroidism (3.3%, 0%). In the non-COPD group, the corresponding adverse events were leukopenia (6.8%, 6.8%), neutropenia (3.3%, 6.8%), fatigue (2.3%, 0%), gastrointestinal reactions (2.3%, 0%), and hypothyroidism (2.3%, 0%), respectively.

**Conclusions:**

The present study indicates that the presence of COPD may improve PCR, prolong PFS, and have an acceptable safety profile in NSCLC patients receiving neoadjuvant ICIs combined with chemotherapy.

## Background

Lung cancer is the second most common malignancy and has the highest tumor-related mortality rate worldwide [1]. Non-small cell lung cancer (NSCLC) is the main pathological type of lung cancer, accounting for approximately 80% of cases [2]. Radical surgery is the main treatment to improve the prognosis of patients with stage I–III resectable NSCLC and the 5-year survival rate of patients ranging from 92% in stage IA to 26% in stage IIIB disease [3]. Unfortunately, approximately 30–55% of patients who undergo radical resection experience disease recurrence, predominantly distant metastases [4].

While the application of neoadjuvant chemotherapy improves the 5-year survival rates of patients, its benefits are limited; for example, survival rate is increased by only approximately 5% [5]. Immune checkpoint inhibitors (ICIs), such as programmed cell death protein 1/programmed death-ligand 1 (PD-1/PD-L1) inhibitors, are effective therapeutic agents that inhibit the immune escape of tumor cells and enhance the antitumor activity of T cells [6, 7]. Several phase III randomized controlled trials (RCTs) have shown that neoadjuvant immunotherapy combined with chemotherapy can improve the pathological responses of patients with NSCLC and show promising improvements in survival [8, 9].

The immune microenvironment constitutes a complex ecosystem comprising heterogeneous cancer cells, invasive immune cells, and stromal cells, which collectively govern both tumor development and the response to therapy [10]. This microenvironment can be affected by abnormal inflammatory responses, such as those caused by a systemic inflammatory disease like chronic obstructive pulmonary disease (COPD) [11, 12]. Mark et al. reported that COPD alters the immune cell composition in surgical tumor specimens, potentially affecting immune effectiveness [13]. Retrospective studies have shown that patients with advanced NSCLC with COPD benefit more from immunotherapy than those without COPD [13–16]. However, the immune microenvironments differ between early and advanced stages of lung cancer [10]. In addition, abnormal inflammatory responses in patients with COPD may increase treatment-related adverse reactions [17]. Thus, it remains unclear whether patients with NSCLC and COPD benefit from neoadjuvant ICIs treatment in combination with chemotherapy.

This study investigated the impact of COPD on the efficacy and safety of neoadjuvant ICIs therapy combined with chemotherapy in patients with resectable NSCLC.

## Methods

### Study participants

This retrospective cohort study analyzed 105 patients with NSCLC who received neoadjuvant immunotherapy combined with chemotherapy at the Department of Thoracic Surgery of Tianjin Chest Hospital between April 2020 and April 2023. Data were extracted from the hospital information system of Tianjin Chest Hospital. The predefined inclusion criteria were as follows: 1) age > 18 years; 2) Eastern Cooperative Oncology Group (ECOG) scores of 0 or 1; 3) pathologically and radiographically confirmed NSCLC with clinical stages IA–IIIB (T1‑T4N2M0, T3‑T4N1M0, or T4N0M0), according to the American Joint Committee on Cancer’s eighth edition clinical staging of lung cancer guidelines; and 4) patients who underwent treatment with ICIs + chemotherapy as a neoadjuvant therapy.

**Fig. 1:**
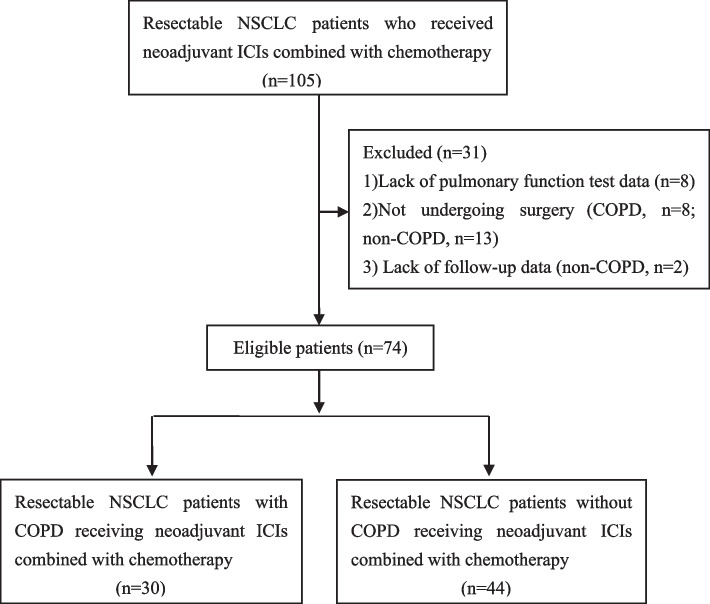
Patient screening and inclusion flow chart

A total of 31 patient records were excluded based on the following predefined exclusion criteria: 1) lack of pulmonary function test data, as the data from other hospitals were not included in the hospital information system of our hospital; 2) absence of surgical intervention post-neoadjuvant immunotherapy combined with chemotherapy due to factors such as disease progression, refusal of surgery, comorbidities, complications, poor lung function, treatment-related toxicity, and unknown reasons; and 3) lack of follow-up data. According to the spirometric criteria of the Global Initiative for Chronic Obstructive Lung Disease (GOLD), COPD was defined as a ratio of forced expiratory volume in 1 s to forced vital capacity (FEV1/FVC) of less than 0.7 using post-bronchodilator spirometry [18]. Finally, this study included 74 patients: 30 with COPD and 44 without. The flowchart depicting patient screening and inclusion is presented in Fig. 1.

### Data collection

Patient information on sex, age, smoking index, Eastern Oncology Cooperative Group Performance score (ECOG PS) score, histology, lung function, clinical TNM (cTNM) stage, and neoadjuvant and adjuvant therapy was collected (if available) by using self-designed data collection tables.

### Outcomes and toxicities

Each surgical specimen from the patients included in this study was re-evaluated for pathological response by two experienced pathologists. Major pathological response (MPR) was defined as the presence of ≤ 10% residual viable carcinoma cells in the primary tumor and sampled lymph nodes, while pathological complete response (PCR) was defined as the presence of 0% residual viable tumor cells in the primary tumor and sampled lymph nodes [8, 9]. The response results based on the Response Evaluation Criteria in Solid Tumors (RECIST, version 1.1) criteria were classified as complete response (CR), partial response (PR), stable disease, or progressive disease (PD). The objective response rate (ORR) was defined as the proportion of CR and PR [19].

All follow-up data were collected until August 2023. Progression-free survival (PFS) was defined as the time from lung cancer diagnosis to disease recurrence, death from any cause, or the last follow-up. All toxicities were documented and graded according to the Common Terminology Criteria for Adverse Events (CTCAE), version 4.03 [20].

### Determination of sample size

Retrospective studies showed that in patients with advanced NSCLC receiving immune checkpoint inhibitors combined with chemotherapy, the Hazard Ratio (HR) associated with PFS in patients with COPD was about 0.5 compared with that in patients without COPD. Additionally, several phase III RCTs have suggested that the PCR in NSCLC patients receiving neoadjuvant immune checkpoint inhibitors combined with chemotherapy is approximately 20%. Given that the prevalence of non-COPD is higher than COPD, it is hypothesized that the ratio of COPD to non-COPD patients included in this study was 1:1.5. The power was set at 0.8 and the significance level was 0.05. The sample size was estimated based on the above parameters by a third-party professional statistician using PASS 2021 software. The results showed that 29 patients should be recruited in the COPD group and 44 in the non-COPD group.

### Statistical analysis

IBM SPSS Statistics for Windows, version 22.0, was used for statistical analysis. Mean and standard deviation (SD) values are used to describe continuous data. Student's *t*-test was used to assess the differences between the COPD and non-COPD groups if the original data followed a normal distribution; otherwise, the Mann–Whitney *U* test was applied. Categorical variables are expressed as numbers (percentages), and the differences between the two groups were compared using chi-square or Fisher exact tests.

The cumulative PFS values were compared between the two groups using Kaplan–Meier (K–M) curves and log-rank tests. Variables that were clinically significant or with *P* < 0.1 in the univariate analysis were included in the multivariate analysis. Both univariate and multivariate analyses of PCR and PFS were performed using logistic and Cox regression models, respectively. *P* < 0.05 indicated a statistically significant difference.

## Results

### Patient characteristics

The baseline patient data are shown in Table 1.

**Table 1:** Patient characteristics and baseline data

	COPD (*n* = 30)	Non-COPD (*n* = 44)	*P*-value
**Age**, Mean ± SD	63.87 ± 5.87	63.14 ± 7.54	0.641
**Sex, n (%)**			0.914
Male	27(90)	38(86.4)	
Female	3(10)	6(13.6)	
**Smoking status, n (%)**			0.982
Never	7(23.3)	11(25)	
Former	6(20)	9(20.5)	
Current	17(56.7)	24(54.5)	
**Smoking index**, Median(range)	35(0–150)	30(0–90)	0.405
**ECOG PS score, n (%)**			0.058
0	9(30)	23(52.3)	
1	21(70)	21(47.7)	
**Histology, n (%)**			0.815
Squamous cell carcinoma	26(86.7)	36(81.8)	
Non -squamous cell carcinoma	4(13.3)	8(18.2)	
**cTNM Stage, n (%)**			0.135
IA	1(3.3)	0(0)	
IB	5(16.7)	4(9.1)	
IIA	2(6.7)	0(0)	
IIB	5(16.7)	9(20.5)	
IIIA	11(36.7)	26(59.1)	
IIIB	6(20)	5(11.4)	
**cT stage, n (%)**			0.941
cT1	5(16.7)	6(14)	
cT2	15(50)	22(50.0)	
cT3	6(20)	8(18.6)	
cT4	4(13.3)	8(18.6)	
**cN stage, n (%)**			0.301
cN0	13(44.8)	12(27.3)	
cN1	3(10.3)	8(18.6)	
cN2	14(46.7)	24(55.8)	
**GOLD grade, n (%)**			
I	8(26.7)	-	
II	21(70)		
III	1(3.3)	-	
**Neoadjuvant cycle, n (%)**			0.369
1 cycle	1(3.3)	0(0)	
2 cycle	17(56.7)	25(56.8)	
3 cycle	12(40)	16(36.4)	
4cycle	0(0)	3(6.8)	
**Treatment regimens, n (%)**			0.978
PD-L1 + chemotherapy	5(16.7)	6(13.6)	
PD-1 + chemotherapy	25(83.3)	38(86.4)	
**Adjuvant cycle, n (%)**			0.966
≤ 6 cycle	21(70)	31(70.5)	
> 6 cycle	9(30)	13(29.5)	

*Abbreviations*: *SD* standard deviation, *ECOG PS* Eastern Cooperative Oncology Group performance status, *GOLD* Global Initiative for Chronic Obstructive Lung Disease

The baseline characteristics, including age; sex; smoking status; ECOG PS score; histological type; neoadjuvant cycle; adjuvant cycle; and cT, cN, and cTNM stage, did not differ between the two groups (all *P* > 0.05). The predominant ECOG scores in the COPD and non-COPD groups were 1 (70%, 21/30) and 0 (52.3%, 23/44), respectively. Squamous cell carcinoma was the main pathological type in both groups (COPD: 86.7%, 26/30 patients; non-COPD: 81.8%, 36/44 patients). The cTNM stage in both groups was mainly stage IIIA (COPD: 36.7%, 11/30 patients; non-COPD: 59.1%, 26/44 patients). Both groups predominantly received two neoadjuvant cycles (COPD: 56.7%, 17/30 patients; non-COPD: 56.8%, 25/44 patients).

### Treatment information

All included patients received neoadjuvant immune checkpoint inhibitors (PD-1 inhibitors and PD-L1 inhibitors) combined with chemotherapy: 25 and 5 patients, respectively, in the COPD group, and 38 and 6 patients, respectively, in the non-COPD group. The chemotherapy regimens were tailored based on tumor pathology, with pemetrexed administered in adenocarcinoma patients and taxane drugs administered for other pathologic types. Pembrolizumab, the most common PD-1 inhibitor, was administered in 10 patients in the COPD group and 19 patients in the non-COPD group. Duvalumab, the most widely used PD-L1 inhibitor, was administered in 4 patients in both the COPD and non-COPD groups. Detailed data are shown in Table 2.

**Table 2:** Summary of neoadjuvant therapy

**Treatment regimens**	COPD	Non-COPD
Pembrolizumab + Paclitaxel liposomes + platin	10	19
Pembrolizumab + nab‑paclitaxel + platin	1	3
Pembrolizumab + Pemetrexed + platin	2	0
Nivolumab + Paclitaxel liposome + platin	2	0
Tirellizumab + Paclitaxel liposome + platin	5	8
Toripalimab + + Paclitaxel liposome + platin	1	1
Sintilimab + Paclitaxel liposome + platin	3	2
Sintilimab + Pemetrexed + platin	0	1
Camrelizumab + Pemetrexed + platin	1	4
Durvalumab + Paclitaxel liposome + platin	4	2
Durvalumab + Pemetrexed + platin	0	2
Atezolizumab + nab‑paclitaxel + platin	1	1
Atezolizumab + Pemetrexed + platin	0	1

### Surgery summary

Among patients who received neoadjuvant ICIs combined with chemotherapy, 21.1% (8/38) of patients with COPD and 22.8% (13/57) of patients without COPD did not undergo definitive surgery. A complete summary of the canceled surgery profiles is presented in Table 3.

**Table 3:** Summary of cancelled surgery

Patients with cancelled definitive surgery-no	COPD	Non-COPD
Disease progressive	2	3
Refusal of surgery	2	4
Comorbidity	1	3
Complication	0	1
Poor lung function	2	0
Treatment-related toxicity	0	1
Unknown reason	1	1

**Fig. 2:**
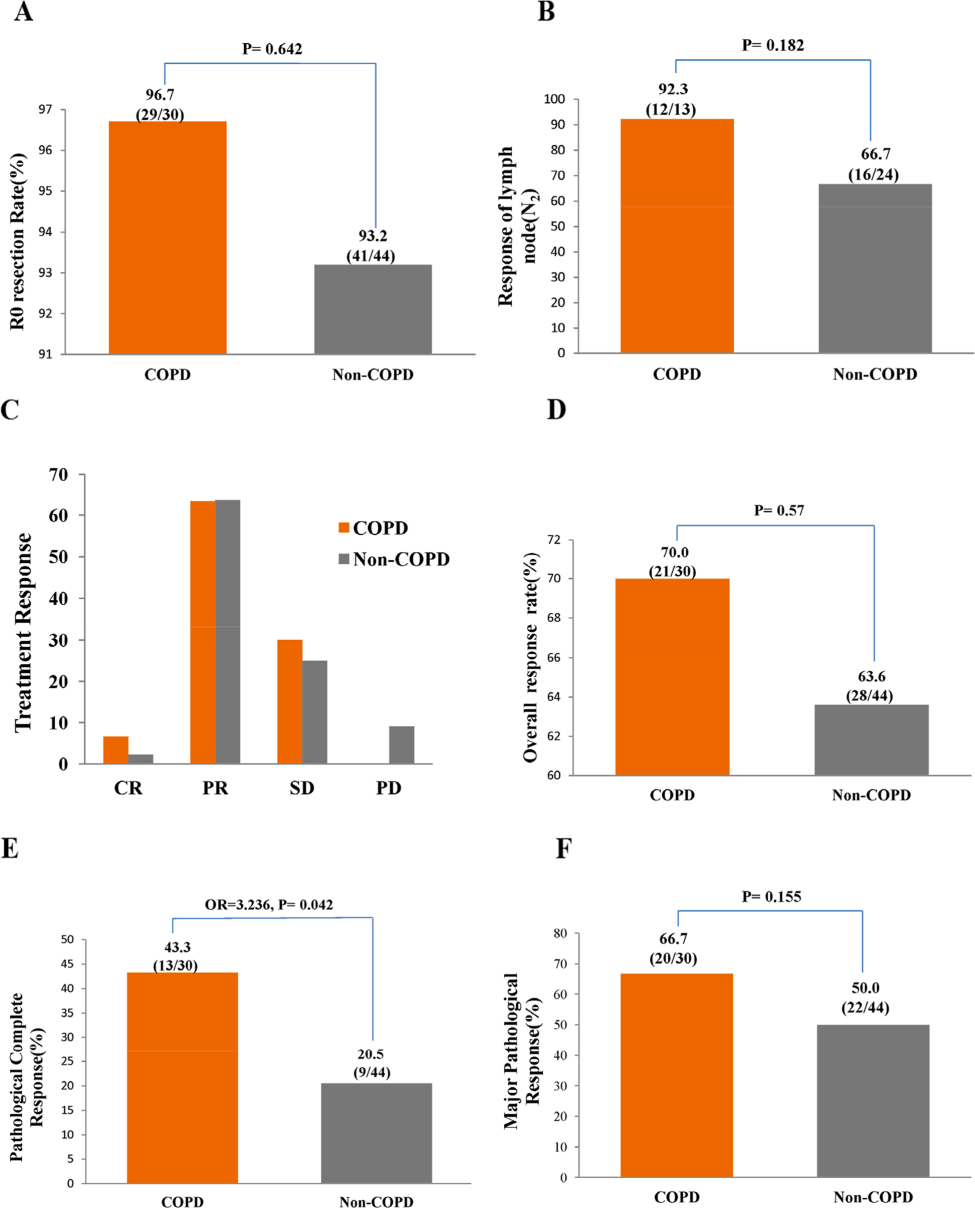
Comparison of (**A**) R0 resection rate, (**B**) Response of lymph node(N2), (**C**) Treatment response, (**D**) Objective response rate, (**E**) Pathological complete response and (**F**) Major pathological response of NSCLC patients receiving neoadjuvant ICI combined with chemotherapy between the COPD and the non-COPD group

Lobectomy and pneumonectomy were the main surgical methods used in both groups undergoing pneumonectomy (COPD: 10%, 3/30 patients; non-COPD: 13.6%, 6/44 patients). R0 resection (no residual tumor) was performed in 96.7% (29/30) of the patients in the COPD group and 93.2% (41/44) of those in the non-COPD group (Fig. 2A).

### Efficacy

**Fig. 3:**
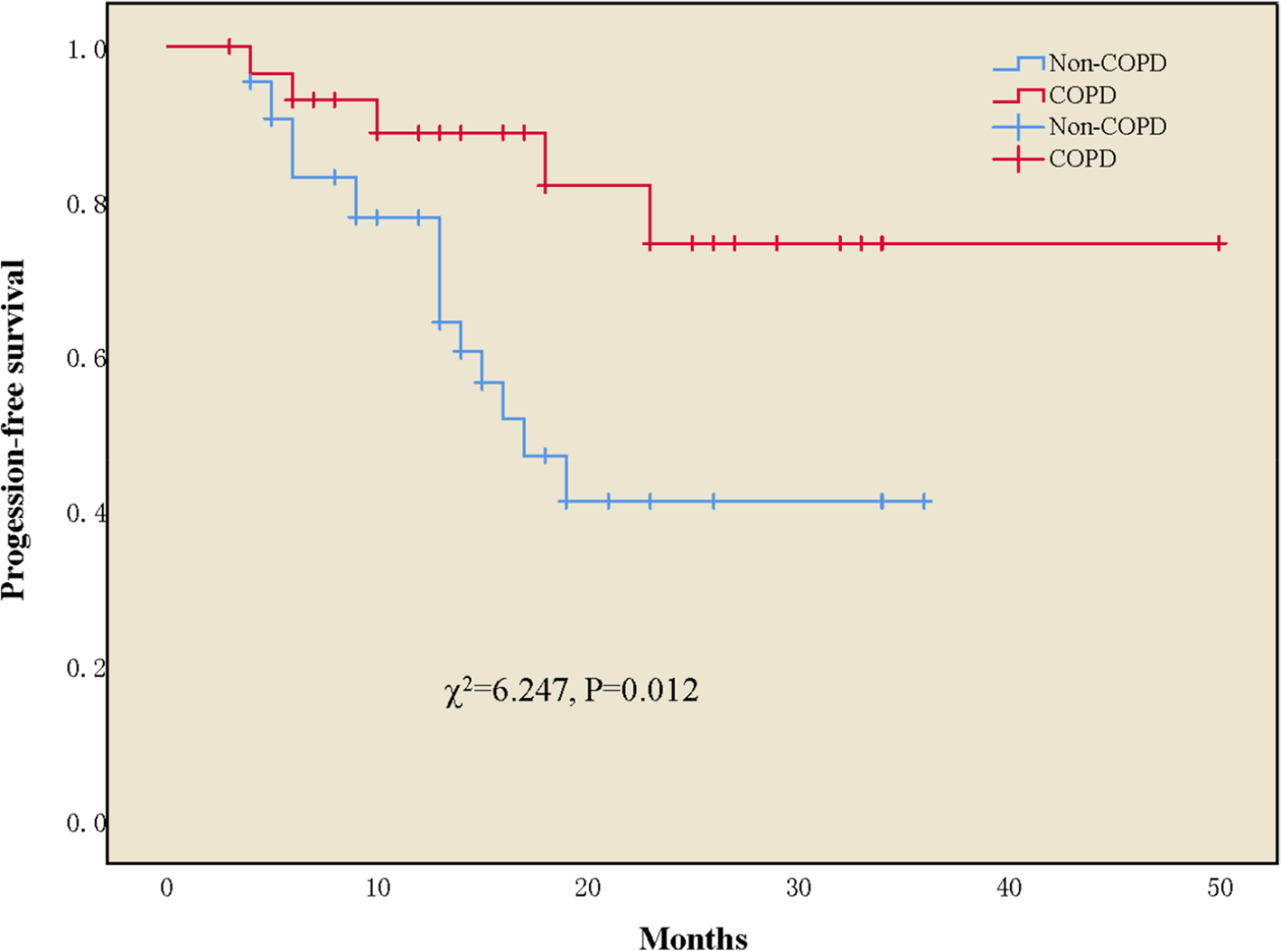
Kaplan–Meier survival curves for overall survival Progression-free survival (PFS)

The incidence of N2 lymph node downstaging (N2 to N1 or N0) was higher in the COPD group than in the non-COPD group (92.3%, 12/13 patients vs. 66.7%, 16/24 patients, *P* = 0.182) (Fig. 2B). CR, PR, SD, and PD were achieved by 2, 19, 9, and 0 patients, respectively, in the COPD group and by 1, 28, 11, and 4 patients, respectively, in the non-COPD group (Fig. 2C). The ORR was higher in the COPD group than in the non-COPD group, but not significantly (70% vs. 63.6%, *P* = 0.57) (Fig. 2D). The percentage of patients with a PCR was higher in the COPD group than in the non-COPD group (43.3% vs. 20.5%, *P* = 0.042) (Fig. 2E). Although not statistically significant, the percentage of patients with a major pathological response was higher in the COPD group than that in the non-COPD group (66.7% vs. 50%, *P* = 0.155, Fig. 2F). With a median follow-up of 18 months, the median PFS was not reached in the COPD group, whereas it was 17 months (95% confidence interval [CI], 12.1–21.9) in the non-COPD group, representing a statistically significant difference (χ^2^ = 6.247, *P* = 0.012, Fig. 3).

The univariate analysis indicated that only the presence of COPD (*P* = 0.038) was a significant factor for PCR. In the multivariate logistic regression analysis of factors associated with PCR, after adjusting for the other factors of clinical significance (smoking index, histology, cTNM stage, and neoadjuvant cycle), COPD remained a significant independent factor (adjusted OR = 3.020, 95%CI: 1.042–8.757; *P* = 0.042, Table 4). In the univariate analysis of factors associated with progression-free survival, the presence of COPD (*P* = 0.020) and cTNM stage (*P* = 0.077) were identified as significant influencing factors. Additionally, the survival analysis results based on the multivariate Cox proportional hazard model showed that after adjusting for cTNM stage and the other clinically significant factors (age, PCR, histology, and neoadjuvant cycle), the adjusted hazard ratio (HRadj) of COPD was statistically significant (HRadj = 0.321, 95%CI: 0.111–0.930; *P* = 0.036, Table 5).

**Table 4:** Factors associated with pathological complete response (PCR) by univariate and multivariate logistic regression analysis

	**Univariate analysis**			**Multivariate analysis**		
	**OR**	**95% CI**	***P*-value**	**OR**	**95% CI**	***P-*value**
Presence of COPD	2.974	1.063–8.318	0.038	3.020	1.042–8.757	0.042
Age(> 65 vs. ≤ 65)	1.333	0.487 -3.654	0.576			
Sex(male vs. female)	1.556	0.297 -8.160	0.601			
SI, pack-years (> 20 vs. ≤ 20)	1.571	0.549 -4.501	0.400	1.328	0.440–4.002	0.615
ECOG PS score (1 vs. 0)	1.500	0.538–4.183	0.438			
Histology(Non-SCC vs. SCC)	0.754	0.183–3.102	0.696	0.897	0.195–4.135	0.889
cTNM Stage^**a**^	1.339	0.465 -3.854	0.588	1.445	0.441–7.731	0.543
Neoadjuvant cycle(> 2 vs. ≤ 2)	1.230	0.450–3.362	0.686	1.222	0.405–3.689	0.722

*Abbreviations*: *SI* Smoking index, *OR* Odds ratio

^a^Stage IA + IIA + IIB vs. IIIA + IIIB

**Table 5:** Factors associated with progression-free survival (PFS) by univariate and multivariate Cox's proportional hazards regression analysis

	**Univariate analysis**			**Multivariate analysis**		
	**HR**	**95% CI**	***P*-value**	**HR**	**95% CI**	***P*-value**
Presence of COPD	0.304	0.112–0.827	0.020	0.321	0.111–0.930	0.036
Age(> 65 vs. ≤ 65)	1.801	0.790 -4.105	0.162	2.733	1.119–6.677	0.027
Sex(male vs. female)	0.770	0.227 -2.616	0.676			
SI, pack-years (> 20 vs. ≤ 20)	0.847	0.357 -2.008	0.705			
PCR	0.675	0.409–1.112	0.123	0.733	0.439–1.225	0.236
Histology(non-SCC vs. SCC)	1.143	0.388–3.371	0.809	0.981	0.302–3.190	0.974
ECOG PS score (1 vs. 0)	1.056	0.463 -2.412	0.896			
cTNM Stage^**a**^	2.448	0.907–6.603	0.077	2.606	0.905–7.508	0.076
Neoadjuvant cycle(> 2 vs. ≤ 2)	1.212	0.504–2.914	0.667	0.937	0.359–2.446	0.895
Adjuvant cycle(> 6vs. ≤ 6)	0.737	0.301–1.803	0.503			

*Abbreviations*: *SI* Smoking index, *HR* Hazard ratio

^a^Stage IA + IIA + IIB vs. IIIA + IIIB

### Adverse events

Three surgery-related adverse events were observed in the COPD group: sustained air leakage into the pleural cavity postoperatively, postoperative acute exacerbation of COPD with type II respiratory failure, and intraoperative cardiac arrest. In the non-COPD group, two surgery-related adverse events occurred: severe postoperative infection and persistent air leakage in the pleural cavity after surgery.

Treatment-related adverse events occurred in 86.7% of 30 patients in the COPD group and 81.8% of 44 patients in the non-COPD group. Twenty percent of patients in the COPD group and 20.5% of those in the non-COPD group had grade 3 or 4 treatment-related adverse events. The most common treatment-related adverse event in both groups was hematological toxicity, particularly leukocytopenia. Non-hematotoxic adverse reactions included fatigue, gastrointestinal reaction, rash, alopecia, elevated liver transaminase, hypothyroidism, and abnormal myocardial enzymes, among which fatigue (33.3% vs. 29.5%) and gastrointestinal reactions (26.7% vs. 22.7%) were the main non-hematotoxic adverse reactions. Interstitial pneumonia was not observed in either group. The grade 3 and grade 4 adverse events in the COPD group were leukopenia (3.3%, 6.7%), neutropenia (3.3%, 6.7%), fatigue (6.7%, 0%), gastrointestinal reactions (3.3%, 0%), and hypothyroidism (3.3%, 0%). In the non-COPD group, the corresponding adverse events were leukopenia (6.8%, 6.8%), neutropenia (3.3%, 6.8%), fatigue (2.3%, 0%), gastrointestinal reactions (2.3%, 0%), and hypothyroidism (2.3%, 0%), respectively. The complete adverse events profiles are presented in Table 6.

**Table 6:** Treatment-related adverse events (unit: cases)

**Treatment-related adverse events-no(%)a**	ALL		Grade 3		Grade 4	
	COPD	Non-COPD	COPD	Non-COPD	COPD	Non-COPD
Fatigue	10(33.3)	13(29.5)	2(6.7)	1(2.3)	0	0
Gastrointestinal reaction	8(26.7)	10(22.7)	1(3.3)	1(2.3)	0	0
Leukocytopenia	13(43.3)	18(40.9)	1(3.3)	3(6.8)	2(6.7)	3(6.8)
Anemia	5(16.7)	8(18.2)	0	0	0	0
Neutropenia	6(20.0)	10(22.7)	1(3.3)	1(3.3)	2(6.7)	3(6.8)
Rash	3(10.0)	1(2.3)	0	0	0	0
Hypothyroidism	2(6.7)	3(6.8)	1(3.3)	1(2.3)	0	0
Elevated transaminase	3(10.0)	5(11.4)	0	0	0	0
Alopecia	3(10.0)	6(13.6)	0	0	0	0
Abnormal myocardial enzyme	1(3.3)	0	0	0	0	0

^a^Included are events reported between the first neoadjuvant dose and 30 days after the last neoadjuvant dose

## Discussion

To our knowledge, this is the first clinical report to evaluate the effect of COPD on the efficacy and safety of neoadjuvant ICIs therapy in combination with chemotherapy in resectable NSCLC. In patients receiving neoadjuvant immunotherapy combined with chemotherapy, compared to patients without COPD, the presence of COPD was associated with a significantly longer PFS (not reached vs. 17 months) and a higher PCR rate (43.3% vs. 20.5%). Multivariate logistic and Cox regression analyses also suggested that COPD was an independent factor associated with the efficacy of neoadjuvant ICIs combined with chemotherapy in patients with NSCLC. Moreover, the adverse event profile showed that COPD did not increase the toxicity of neoadjuvant ICIs in combination with chemotherapy.

Compared with neoadjuvant chemotherapy, neoadjuvant ICIs combined with chemotherapy can significantly improve the PCR, EFS, and overall survival of patients with NSCLC [8, 9, 21, 22]. However, no studies focused on COPD, a common comorbidity of NSCLC, which may be related to the efficacy and toxicity of immune checkpoint inhibitors [13–17]. Our study investigated the efficacy and safety of neoadjuvant immunotherapy combined with chemotherapy in patients with NSCLC and COPD. Several phase III RCTs have demonstrated PCR rates of 17.2–24% in patients receiving neoadjuvant immunotherapy combined with chemotherapy for NSCLC [8, 9, 21]. Similarly, the results of this study showed that the PCR rate of patients with NSCLC without COPD was 20.5%, while the PCR rate of NSCLC patients with COPD was even higher at 43.3%. In addition to PCR and PFS, the COPD group also showed superior MPRs, R0 resection rates, and N2 lymph node downstaging rates compared to the non-COPD group, indicating that patients with COPD could benefit more from neoadjuvant ICIs combined with chemotherapy.

The present study suggests that COPD can significantly affect the efficacy of neoadjuvant immunotherapy. One explanation may be that COPD alters the immune microenvironment of tumors independent of smoking, which may also affect the immune microenvironment [13, 23]. Previous studies have shown an increase in T-helper cell type 1 (Th1) differentiation of lymphocytes within the tumor microenvironment of NSCLC patients with COPD [13, 24]. Biton et al. revealed that the presence of COPD can up-regulate the expression of PD-1 and TIM3 on the surface of CD8 + T lymphocytes, resulting in the depletion of infiltrating T lymphocytes in the tumor microenvironment [24]. Furthermore, COPD also causes changes in the ratio of Treg/Th17 cells, a decrease in granulocyte myeloid-derived suppressor cells, and an increase in PD-1^+^ tumor-associated macrophages [11, 16, 25]. In addition to the changes in the tumor immune microenvironment, an increased expression of PD-L1 in tumor cells [11, 26], promoter methylation of *CTLA4*, *LAG3*, and *PD-L1*, and an abnormal expression of microRNAs [27–29] can affect the efficacy of ICIs in NSCLC patients with COPD.

The presence of driver genes poses a challenge for immunotherapy in advanced NSCLC [30]. In this study, all patients with non-squamous cell carcinoma tested negative for driver genes, whereas most patients with squamous cell carcinoma were not tested. The Chinese Society of Clinical Oncology guidelines do not recommend routine genetic testing for squamous cell carcinoma because of the low probability of genetic mutations [31]. Given the low cost–benefit ratio of genetic testing, most patients do not undergo genetic testing. In addition, patients who are positive for driver genes can benefit from neoadjuvant immunotherapy in combination with chemotherapy [9, 32]. Although the present study did not consider data on driver genes, this likely had a limited impact on the conclusions of the study.

COPD is a systemic inflammatory disease, and its associated abnormal inflammatory response affects not only the immunotherapy efficacy but also immune-related adverse events (irAEs). Zhang et al. found that the incidence of irAEs in patients with advanced NSCLC with COPD was associated with the GOLD grade, while the incidence in patients with mild-to-moderate COPD was similar to that in patients without COPD [17]. In the present study, we found that the types and rates of treatment-related adverse events were similar in both the COPD and non-COPD groups, suggesting that COPD does not increase the toxicity of neoadjuvant immunotherapy. One possible reason is that most patients with COPD in this study had a lung function rating of GOLD I or II, with only one case of GOLD III. In addition, this study explored the impact of COPD on the safety of neoadjuvant therapy, with a limited duration. ICI-related pneumonitis (CIP) is a life-threatening irAE that causes extensive respiratory symptoms and parenchymal abnormalities in the lungs, leading to respiratory failure. In the present study, CIP was not observed in either patient group. Currently, the impact of COPD on the incidence of CIP in patients with advanced NSCLC is controversial, and whether it affects the incidence of CIP during neoadjuvant immunotherapy remains unclear [14, 33, 34].

Our study has some limitations. First, as a retrospective observational study based on data from medical records, unexpected confounding biases and the influence of data deficiencies could not be excluded. Second, PD-L1 expression level is a major predictive biomarker for immunotherapy in advanced NSCLC, but its role in neoadjuvant immunotherapy is unclear [8, 35]. At present, PD-L1 detection is not necessary for patients receiving neoadjuvant immunotherapy, and only 5 patients in this retrospective study were tested for PD-L1. Third, the pathological type in most patients in this study was squamous cell carcinoma, whereas few patients had adenocarcinoma. Therefore, whether the conclusions of this study are applicable to patients with adenocarcinoma is unclear, and further research is needed.

## Conclusion

The results of the present study indicated that the presence of COPD may improve PCRs, prolong PFS, and maintain an acceptable safety profile in patients with NSCLC receiving neoadjuvant ICIs in combination with chemotherapy. Neoadjuvant ICI therapy combined with chemotherapy is feasible in patients with resectable NSCLC with COPD. However, further studies are required to provide additional evidence to support this finding.

## Data Availability

The original data can be acquired from the corresponding authors under reasonable requirement.

## Abbreviations

ICIs: Immune checkpoint inhibitors
COPD: Chronic obstructive pulmonary disease
FEV1: Forced expiratory volume in 1 s
FVC: Forced vital capacity
NSCLC: Non-small cell lung cancer
CR: Complete response
PR: Partial response
SD: Stable disease
ORR: Objective response rate
PCR: Pathological complete response
MPR: Major pathological response
PFS: Progression-free survival
ECOG PS: Eastern Oncology Cooperative Group Performance status
SI: Smoking index
SD: Standard deviation
CI: Confidence interval
CIP: ICI-related pneumonitis
GOLD: Global Initiative for Chronic Obstructive Lung Disease
